# Prognostic Value of the CONUT Score in Predicting All-Cause Mortality in Hospitalized Internal Medicine Patients: A Retrospective Cohort Study

**DOI:** 10.3390/jcm15051904

**Published:** 2026-03-02

**Authors:** Betül Çavuşoğlu Türker, Mehmet Yamak, Mehmet Çetin, Serkan Çakır, Özlem Menken, Pınar Doğan, Vildan Söğüt Karayigit, Alihan Oral, Fatih Türker

**Affiliations:** 1 Department of İnternal Medicine, University of Health Sciences Turkey, Haseki Health Training and Research Hospital, Aksaray, Dr. Adnan Adıvar Cd. No: 9, 34130 Istanbul, Turkey; m-yamak@hotmail.com (M.Y.); dr.cetin35@outlook.com (M.Ç.); pinar.kulahli@gmail.com (P.D.); vildansogut@gmail.com (V.S.K.); fatihturker1985@hotmail.com (F.T.); 2 Department of Internal Medicine, Bayburt State Hospital, 69000 Bayburt, Turkey; serkancakir724@hotmail.com; 3 Department of Internal Medicine, Defne State Hospital, 31000 Hatay, Turkey; ozlemmenken@gmail.com; 4 Department of Internal Medicine, Faculty of Medicine, Biruni University, 34015 Istanbul, Turkey; dr.alihanoral@gmail.com

**Keywords:** CONUT score, mortality

## Abstract

**Aim:** This study aimed to evaluate the prognostic significance of the CONUT score and its association with all-cause mortality in hospitalized internal medicine patients. **Methods:** This retrospective cohort study included hospitalized adult patients followed for long-term all-cause mortality. Demographic data, laboratory parameters, comorbidities, and CONUT scores were recorded at admission. The CONUT score was calculated using serum albumin, total cholesterol, and lymphocyte count. Survival analysis was performed using the Cox proportional hazards regression model. Variables with *p* < 0.1 in univariate analysis were entered into the multivariate model. Hazard ratios (HRs) and 95% confidence intervals (CIs) were calculated. The primary outcome was all-cause mortality. **Results:** During the follow-up period, the CONUT score showed a strong and significant association with mortality. In multivariate Cox regression analysis, age, CONUT score, and chronic renal disease were identified as independent predictors of all-cause mortality. Each one-year increase in age was associated with a 5.3% increase in mortality risk (HR = 1.053, 95% CI: 1.048–1.058, *p* < 0.001). Each one-point increase in CONUT score nearly doubled the risk of death (HR = 1.219, 95% CI: 1.190–1.250, *p* < 0.001). The presence of chronic renal failure (HR = 2.142, *p* < 0.001) and solid organ malignancy (HR=1.216 *p* < 0.001) significantly increased mortality risk. **Conclusions:** The CONUT score is a powerful and independent predictor of all-cause mortality in hospitalized internal medicine patients. As a simple, inexpensive, and routinely available tool, CONUT can be easily integrated into daily clinical practice for early risk stratification and identification of high-risk patients who may benefit from closer monitoring and nutritional intervention.

## 1. Introduction

Internal medicine clinic services serve the aging patient population who have many chronic diseases. Older inpatients have a high incidence of malnutrition [1], which is one of several adverse factors affecting mortality risk in hospitalized patients and may be able to predict adverse clinical outcomes, such as frailty, immune dysfunction, anemia, reduced cognitive function, length of hospitalization, and mortality [2]. Providing nutritional interventions to malnourished inpatients is known to reduce patients’ length of hospital stay, the need for readmission, and costs and to improve cognitive, physical, and social functionality [3,4]. As a result, appropriate screening of nutritional status and assessing malnutrition status are very important for all hospitalized patients. Current studies have shown no gold standard method to exist for diagnosing malnutrition, which makes diagnosing malnutrition difficult, as many nutritional scores need to be used to determine inpatients’ nutritional status [5].

The CONUT score was first developed by Ignacio et al. in 2005 as a practical tool designed to monitor the nutritional status of hospitalized patients [6]. The Controlling Nutritional Status (CONUT) score is calculated from serum albumin, total cholesterol, and total lymphocyte levels, and recent studies have determined it to be a simple and efficient screening tool for evaluating malnutrition status in hospitalized patients [7,8]. The CONUT score has been recommended as an immune–nutritional index for assessing both protein/lipid metabolism and immunocompetence. The score essentially reflects protein status, caloric deficiency, and immune competence through serum albumin, total cholesterol, and lymphocyte count, respectively [9,10]. Evaluating nutritional status is vital in the care of chronic disease patients. Moreover, CONUT is associated with short- and long-term prognoses in some diseases, such as malignancies, cardiovascular disease, and stroke [11,12]. Liu et al. demonstrated high CONUT scores to be associated with an increased risk of in-hospital mortality, short-term complications, and longer hospital stays in older adult patients [13].

Therefore, this study aims to evaluate and explore the association that CONUT scores have with mortality regarding inpatients in an internal medicine clinic.

## 2. Materials and Methods

### 2.1. Ethical Aspects

This study was approved by the Ethics Committee of Haseki Training and Research Hospital, University of Health Sciences, Istanbul, Turkey (Approval No: 209-2025, Date: 5 November 2025). The study was conducted in accordance with the principles of the Declaration of Helsinki and the guidelines of Good Clinical Practice. Written informed consent was obtained from all participants during their hospitalization.

### 2.2. Study Design

This study was designed as an observational, retrospective cohort study and was carried out at the Internal Medicine Clinic of Haseki Training and Research Hospital. Electronic medical records of hospitalized patients between 1 January and 31 December 2019 were used for data collection and analysis.

### 2.3. Study Participants

A total of 1768 hospitalized and discharged patients were included in the study. Patients younger than 18 years of age, pregnant women, those with delayed hospital admission longer than 24 h, patients with hematological malignancies, those using anti-inflammatory drugs or immunosuppressive agents such as steroids, patients who were readmitted during the study period, and those with insufficient clinical or laboratory data were excluded. In addition, patients whose cause of death was due to unnatural reasons, such as accidents, suicide, or homicide, were also excluded. Demographic data, including age and sex, as well as clinical variables such as the presence of diabetes mellitus, hypertension, coronary artery disease, chronic kidney disease, and solid malignancies, were recorded for all participants.

### 2.4. Laboratory Analyses

Laboratory parameters were obtained from fasting morning blood samples collected at the time of hospital admission. These parameters included white blood cell count, neutrophil count, lymphocyte count, hemoglobin, C-reactive protein, serum albumin, total cholesterol, thyroid-stimulating hormone, and ferritin levels. The CONUT score was calculated for each patient by summing the scores derived from serum albumin levels, total cholesterol concentrations, and total lymphocyte counts. According to the total CONUT score, patients were classified into four groups: normal nutritional status (0–1), mild malnutrition (2–4), moderate malnutrition (5–8), and severe malnutrition (9–12).

### 2.5. Follow-Up and Study Outcomes

All follow-up data were obtained from the data processing system of Haseki Training and Research Hospital, and mortality outcomes were verified through the Turkish National Mortality Registry to ensure accuracy. The primary outcome of this study was to evaluate the prognostic value of the Controlling Nutritional Status (CONUT) score in predicting all-cause mortality in hospitalized patients followed in the Internal Medicine clinic.

The secondary outcomes were to investigate the association between the CONUT score and major clinical and laboratory parameters, to compare mortality rates among different CONUT score categories (normal, mild, moderate, and severe), and to determine the discriminative performance of the CONUT score by ROC curve analysis, including the evaluation of the optimal cutoff value for mortality prediction.

### 2.6. Statistical Analysis

Data were expressed as mean ± standard deviation. Statistical analyses were performed using SPSS version 24.0 (SPSS Inc., Chicago, IL, USA). The normality of data distribution was assessed using the Kolmogorov–Smirnov test. Comparisons between two independent groups were performed using the Mann–Whitney U test for continuous variables and the chi-square test for categorical variables, while comparisons among more than two groups were conducted using one-way analysis of variance or the Kruskal–Wallis test as appropriate. Bivariate correlations between continuous variables were evaluated using Pearson’s correlation analysis. A two-tailed *p*-value of less than 0.05 was considered statistically significant. The Cox proportional hazards regression model was used to identify independent predictors of mortality, and survival analyses were performed using the Kaplan–Meier method, with differences between groups compared using the log-rank test.

## 3. Results

This study includes a total of 1,768 patients (929 females, 839 males). The study population had a wide age distribution, ranging from 17.0 to 97.0 years, and the median age was 64 years. The baseline characteristics of the survivor and nonsurvivor groups are presented in Table 1. When compared to the surviving patients, the nonsurvivor patients were older (*p* = 0.001) and had a higher prevalence of diabetes mellitus, hypertension, malignancy, chronic kidney disease and coronary artery disease (*p* < 0.001) (Graph-1). The nonsurvivor group had significantly higher CRP, white blood cell, neutrophil, ferritin, and TSH levels and significantly lower lymphocyte, total cholesterol, hemoglobin, and albumin levels. Compared to the survivor group, the nonsurvivor group exhibited a significantly higher CONUT score (Table 1).

Multivariate Cox regression analysis demonstrated that age, CONUT score, chronic renal failure and solid organ malignancy were independent predictors of all-cause mortality. Each one-year increase in age increased mortality risk by 5.3% (HR = 1.053, 95% CI: 1.048–1.058, *p* < 0.001). Each one-point increase in CONUT score was associated with an almost one-fold increase in mortality risk. In addition, chronic renal failure (HR = 1.670) and solid organ malignancy (HR = 1.216) significantly increased the risk of death (all *p* < 0.001). (Table 2).

The CONUT score demonstrated significant discriminative ability in distinguishing between the survivor and nonsurvivor groups, with an area under the curve (AUC) of 0.739 (95% CI: 0.716–0.762). At a cutoff value of 4, the CONUT score also showed statistically significant discrimination, yielding an AUC of 0.678 (95% CI: 0.653–0.703) (Table 3).

**Figure 1:**
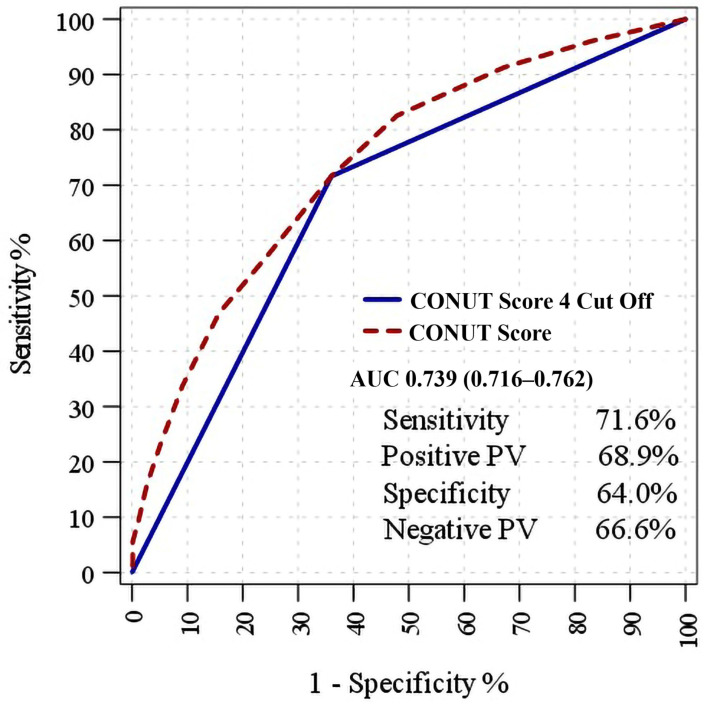
Kaplan–Meier curve for all-cause mortality by CONUT score.

At the cutoff value of 4, the CONUT score had a sensitivity of 71.6%, specificity of 64.0%, positive predictive value of 68.9%, and negative predictive value of 66.9% for differentiating between patients with and without mortality. (Figure 1).

## 4. Discussion

In this study, we investigated the utility and predictive role of the CONUT score in evaluating all-cause mortality for hospitalized patients in a department of internal medicine. While age and major comorbidities, such as diabetes, hypertension, malignancy, and chronic kidney disease, are well-established predictors of mortality, our study demonstrates that the CONUT score provides additional prognostic information beyond these traditional risk factors [14,15].

Apart from traditional risk factors, laboratory-based nutritional indices have been increasingly used for mortality prediction. The CONUT score enables rapid identification of patients at nutritional risk and supports early clinical decision-making regarding nutritional interventions. Furthermore, its prognostic value allows clinicians to stratify patients according to mortality risk and to monitor treatment response over time. Clinically, the CONUT score is cost-effective and easily obtained through routine blood screening. This index can be used to predict mortality in various diseases, and studies on this index are relatively new and limited.

Similar to the current study, Liu et al. identified the CONUT score as a predictive marker of in-hospital mortality in elderly patients [11]. Previous research has indicated that CONUT is associated with prognosis across diverse clinical conditions, including heart failure, chronic liver disease, and malignancies, suggesting its broad applicability as a nutritional and inflammatory marker. Our findings extend this evidence to a heterogeneous internal medicine patient population [16,17]

The CONUT score integrates serum albumin, total lymphocyte count, and total cholesterol, reflecting protein reserves, immune competence, and caloric status, respectively. Moreover, recent studies have demonstrated that albumin and lymphocyte levels reflect chronic inflammation and immune function, which are closely related to nutritional status. Also, mortality is definitely known to increase with malnutrition [2,18]. These nutritional parameters are calculated using objective parameters that are low cost, easy to evaluate, and simple.

Serum albumin level is a component of the CONUT score, with low levels being associated with increased mortality. Mathioudakis et al. showed poor nutritional status, as reflected by lower albumin levels, to be associated with poor prognosis in patients with COVID-19 [19]. Two main mechanisms can be mentioned as the reason for this situation. Firstly, albumin has specific antioxidant properties due to its structure, and hypoalbuminemia can cause increased cellular oxidative damage and apoptosis [20]. Secondly, serum albumin levels provide information regarding systemic protein metabolism and inflammation levels. Studies have also shown low serum albumin levels to perhaps be an associated marker of nutritional status and mortality risk [21].

Recent studies have shown lymphocyte count to be a more stable indicator of body composition during long-term follow-up [2]. Also, lower TLC levels have been associated with lower immune status and higher inflammatory status, as well as with higher mortality rates and poor prognoses in inpatients [22].

Low TC levels represent poor nutritional status, a higher risk of death, and increased inflammation in inpatients [23].

Among the studies included in the meta-analysis, several reported CONUT cutoff values between 2 and 5 as effective predictors of clinical outcomes. Variations in nutritional–metabolic profiles and disease severity across patient phenotypes may explain these differences in optimal thresholds. In our study, the CONUT cutoff value was set at 4. The European Society for Clinical Nutrition and Metabolism (ESPEN) similarly recommends a cutoff value of ≥4 for the diagnosis of malnutrition, which is consistent with our findings; however, this standard still requires validation across diverse ethnic and clinical populations.

A major limitation of our study is its retrospective, single-center design, which may restrict the generalizability of the findings. Additionally, other parameters that could provide a more comprehensive assessment of malnutrition—such as BMI, the Mini Nutritional Assessment (MNA), dietary intake information, smoking status, alcohol consumption, dietary habits and functional status scores—were not available. Since all patients were hospitalized for acute medical conditions, inflammatory markers and nutritional parameters may have been influenced by acute illness, which could represent a potential source of bias.

This study has strong aspects. It is one of the first to investigate the prognostic value of the CONUT score in all-cause mortality in patients hospitalized in an internal medicine clinic. Meanwhile, research on this subject is recent and limited. When evaluating the literature, the number of cases is seen to be small and related to limited indications. The features that distinguish the current study from others are its high number of cases, long follow-up period, and a broad group of diseases.

## 5. Conclusions

In this large retrospective cohort of hospitalized internal medicine patients, the CONUT score was identified as a strong and independent predictor of all-cause mortality, maintaining its prognostic significance even after adjustment for age, major comorbidities, and inflammatory markers, thereby highlighting the critical role of nutritional status in patient outcomes. Owing to its simplicity, low cost, and reliance on routinely available laboratory parameters, the CONUT score represents a practical and easily implementable tool for early risk stratification in daily clinical practice, enabling the timely identification of high-risk patients who may benefit from closer clinical surveillance and early nutritional interventions. These findings emphasize the importance of incorporating nutritional assessment into standard patient evaluation in internal medicine settings and warrant further prospective, multicenter studies to confirm these results and to explore whether CONUT-guided nutritional strategies can improve survival outcomes.

## Figures and Tables

**Table 1:** The baseline characteristics of the survivor and nonsurvivor groups.

	Survivor	NonSurvivor	*p*
Gender: Female n (%) Male n (%)	459 378	470 461	0.067
Diabetes Mellitus, n(%)	265 (31.7%)	403 (43.3%)	0.001
Hypertension, n (%)	342 (40.9%)	589 (63.3%)	≤0.001
Coronary Artery Disease, n (%)	148 (17.7%)	347 (37.3%)	≤0.001
Chronic Kidney Disease, n (%)	90 (10.8%)	285 (30.6%)	≤0.001
Solid malignancy	23 (2.7%)	159 (17%)	≤0.001
Age (Years)	50.6 ± 17.6	71.0 ± 13.4	≤0.001
White blood cell count ×10^9^/L	8.4 ± 3.7	9.5 ± 4.7	≤0.001
Neutrophil ×10^9^/L	5.8 ± 3.4	7.3 ± 4.4	≤0.001
Lymphocyte ×10^9^/L	1.6 ± 0.7	1.2 ± 0.6	≤0.001
Hemoglobin (g/dL)	11.1 ± 2.8	10.1 ± 2.3	≤0.001
Hs-CRP (mg/L)	43.4 ± 75.4	72.6 ± 81.5	≤0.001
Albumin (g/L)	35.5 ± 4.8	30.6 ± 5.7	≤0.001
Cholesterol (mg/dL)	167.5 ± 75.6	158.6 ± 61.9	0.001
TSH (mIU/L)	2 ± 3.4	2.5 ± 7.2	≤0.001
Ferritin (µg/L)	150.1 ± 351.3	246 ± 364.1	≤0.001
CONUT score	2 * (0–12)	5 * (0–12)	≤0.001
Normal (0–1)	277	82	
Mild (2–4)	353	312	
Moderate (5–8)	185	391	
Severe (9–12)	22	146	

Statistically significant variables (*p* < 0.05). * median (min-max). Abbreviations: CRP, C-reactive protein; TSH, Thyroid Stimulating Hormone; CONUT, Controlling Nutritional Status.

**Table 2:** Multivariate Cox proportional hazards regression analysis for all-cause mortality.

	HR	%95 Confidence Interval	*p*-Value
Age	1.053	1.048–1.058	<0.001
CONUT score	1.219	1.190–1.250	<0.001
Solid malignancy	1.216	1.162–1.272	<0.001
Chronic Renal Failure	1.670	1.436–1.943	<0.001

Note: Statistically significant variables (*p* < 0.05). Abbreviations: CONUT, Controlling Nutritional Status.

**Table 3:** ROC analysis of the CONUT score and diagnostic performance of the cutoff value of 4 for predicting all-cause mortality.

		AUC		% 95 Confidence Interval	*p*-Value
CONUT Score		0.739		0.716–0.762	<0.001
CONUT Score 4 Cut Off		0.678		0.653–0.703	<0.001
		Survivor	Nonsurvivor		%
CONUT Score	<4	533	264	Sensitivity	71.6%
	≥4	300	666	Positive Predictive Value	68.9%
				Specificity	64.0%
				Negative Predictive Value	66.9%

## Data Availability

While some or all datasets generated and/or analyzed during the current study are not publicly available, they can be made available from the Ethics Committee of Haseki Training and Research Hospital upon reasonable request.
